# Absorption of Remedies into the Skin

**Published:** 1907-02

**Authors:** 


### ABSORPTION OF REMEDIES INTO THE SKIN.

Dr. Sutton, of Kansas City, Mo., working in Dr. Unna's Clinic in Hamburg, has made a series of observations, of more than general interest, on the absorbability of remedies through the skin (Monatshefte fur praktische Dermatologie, Oct. 15, 1906.)

The problems presented were of unusual difficulty, as it is generally held that not only is the composition of the remedy in question of importance, but the character of the menstruum as well. It has been held for many years by competent pharmacologists that those substances which are capable of volatilization are best capable of being absorbed by the unbroken skin, and of producing general systemic effects, but it is by no means established what conditions in the menstruum are best adapted to further the result desired.

It is to the solution of this side of the problem that Dr. Sutton applied his experimental methods. Lower animals were used, and the method of saturating the menstruum experimented on by a fat, soluble aniline dye was adopted, the staining qualities of the dye being taken as an index to the grade of absorption. After several hours of exposure, pieces of the skin were excised and frozen sections made and studied microscopically. Thus it may be seen that Dr. Sutton limited himself to the problem of local action entirely.

In all, some 200 experiments were performed with some fifty or more different menstrua. Of the dyes used, fuchsine and scharlach R. gave the most satisfactory microscopical pictures. The sebaceous glands showed the effects of the infiltration most markedly, while parts of the hair follicles below the opening of the glands were not affected, thus bearing out the clinical experience of many dermatologists that the lower parts of the hair follicles are reached by remedies with the greatest difficulty.

As to his results, he shows that notwithstanding the well-known energetic disinfecting properties of phenol on the surface of the skin, it has very slight penetrating power. Turpentine and petroleum have about the same properties in this respect. Vaselin and substances of like chemical nature are absorbed very slowly and incompletely. A thorough mixture of the different vehicles with a small quantity of cedar oil seems very materially to hasten their absorption, in spite of the fact that cedar oil alone has no particular power in that direction. Goose fat is one of the best of the penetrating vehicles, but a good pure article is difficult to obtain, and if obtainable its price is almost prohibitive. The sulphon constituents of ichthyol were well absorbed, and mixtures of equal parts of ichthyol with olive oil, or benzol, or goose fat, showed a quicker and more complete absorption of the sulphons than when the artificial sulphons or their ammoniacal combinations were employed.

Sutton shows, in conclusion, that the best absorption for local action is obtained through the media of goose fat, by olive oil, by ichthyol in combination with olive or cedar oil, and by santal oil with or without cedar oil.—Merch's Archives, January, 1907.

